# Chemical, Microbiological, and Functional Characterization of Kefir Produced from Cow's Milk and Soy Milk

**DOI:** 10.1155/2020/7019286

**Published:** 2020-05-12

**Authors:** Raúl Ricardo Gamba, Shihori Yamamoto, Mahmoud Abdel-Hamid, Tetsuya Sasaki, Toshihide Michihata, Takashi Koyanagi, Toshiki Enomoto

**Affiliations:** ^1^Department of Food Science, Ishikawa Prefectural University, Nonoichi, Ishikawa 921-8836, Japan; ^2^Dairy Science Department, Faculty of Agriculture, Cairo University, Giza, Egypt; ^3^Chemistry and Food Department, Industrial Research Institute of Ishikawa, Kanazawa, Ishikawa 920-0223, Japan

## Abstract

Kefir is a functional beverage that contains lactic and acetic acid bacteria (LAB, AAB) and yeasts. This work's aim was to study the chemical, microbial, and functional characteristics of kefir produced from cow's milk and soy milk. After fermentation, free amino acids were 20.92 mg 100 mL^−1^ and 36.20 mg 100 mL^−1^ for cow's milk and soy milk kefir, respectively. Glutamic acid was majority in both, suggesting that microbial proteolysis leads to an increase in free amino acids including glutamic acid. 10^8^–10^9^ CFU mL^−1^ LAB, 10^6^–10^7^ CFU mL^−1^ AAB, and 10^6^–10^7^ CFU mL^−1^ yeasts were counted in cow's milk kefir, whereas soy milk kefir contained greatly lower yeasts and AAB. *Lactococcus lactis*, *Kazachstania unispora*, and *Saccharomyces cerevisiae* were isolated as major microorganisms in both kefirs. *Acetobacter orientalis* only existed in cow's milk kefir. Cow's milk and soy milk showed ACE inhibitory activity, which significantly increased after fermentation. Both kefirs also exhibited antioxidant activity and bactericidal activity against *Escherichia coli*, *Salmonella* Typhimurium, and *Staphylococcus aureus*.

## 1. Introduction

Kefir is a fermented beverage consumed since ancient times that presents typical flavour characteristics through the presence of lactic and acetic acids, ethanol, and carbon dioxide. Several health-promoting properties have been associated with kefir consumption [1], and various properties including antitumor, antibacterial, antioxidant, antifungal, antimutagenic, and hypocholesterolaemia have been demonstrated [2–4]. The main raw material for kefir, cow's milk, is consumed worldwide, with a total production of 770bn litres in 2013, equivalent to USD$328bn, as the top agricultural commodity [5]. However, for various reasons some people do not consume milk: they may be vegan or have health problems such as lactose intolerance or casein allergy. Thereby, soy milk emerges as a potential replacement in kefir fermentation. The consumption of soybeans and their derivatives provides benefits to human health due to the presence of proteins, isoflavones, oligosaccharides, and other constituents [6].

Nowadays, knowledge of natural products with different functional properties is increasing. Cardiovascular disease accounts for approximately 17 million deaths per year worldwide. Within this value, complications of hypertension cause 9.4 million deaths worldwide annually. Hypertension is responsible for at least 45% of deaths due to heart disease and 51% of deaths due to stroke [7]. Angiotensin converting enzyme (ACE) influences blood pressure by converting angiotensin I (AT-I) to AT-II, a strong vasoconstrictor. This enzyme also stimulates aldosterone in the kidneys causing liquid retention in the body, triggering an increase in blood pressure [8]. Therefore, research on foods containing ACE inhibitory compounds has become important in the hope of counteracting this public health problem. Antioxidant activity is another important food function. Although aerobic metabolism creates energy in the body, reactive oxygen species are also produced. These compounds are associated with several diseases such as atherosclerosis, arthritis, cancer, and hypertension [9]. Our bodies have different mechanisms to control these oxygen radicals but they are not completely effective, and intake of foods with additional antioxidants is desirable. Antibacterial activity is also gaining much attention in the field of functional food research due to the problems arising from excessive use of antibiotics. *Escherichia coli* and nontyphoidal *Salmonella* species (such as *S.* Typhimurium) along with *Campylobacter* species and norovirus are mainly responsible for diarrhoeal diseases [10]. Nontyphoidal salmonellae are a leading cause of bacterial diarrhoea worldwide, estimated to cause approximately 153 million cases of gastroenteritis and 57,000 deaths globally each year [11]. We previously reported the ACE inhibitory and antibacterial activities of sugary kefir made from various types of sugar solution, and demonstrated that these activities were significantly enhanced after fermentation [12]. The microbial and chemical compositions of kefir, as well as the way they change during fermentation, are important in determining the above benefits as a functional food. However, detailed published research on kefir is limited, possibly due to the nutritional and microbial diversity of kefir products influenced by multiple factors such as milk composition, origin of the grains, fermentation time and temperature, and storage conditions. The aim of this work was to study and compare the chemical composition, microbial composition, and ACE inhibitory, antioxidant, and antibacterial activities of kefirs fermented from cow's milk and soy milk, for a better understanding of underlying mechanisms and potential health benefits.

## 2. Materials and Methods

### 2.1. Samples and Inoculation of Kefir Grains

Kefir grains CIDCA AGK1 were kindly provided by Professor Graciela De Antoni of National University of La Plata (*Argentina*). The grains were preserved in sterilised skimmed milk (Meiji Holding Co., Ltd., Japan) at −80°C and activated for three successive passages at 25°C for 24 h (10% w/v) in skimmed milk [13]. Soy milk (Kikkoman®, Tokyo, Japan) was bought at a local supermarket. Kefir grains in soy milk were grown over 40 days to ensure their ability to ferment the medium. The media were replaced three or four times a week by fresh medium. For the respective assays, activated grains were used. The grains were incubated with cow's milk or soy milk at 25°C for 24 h. After fermentation, the grains were removed by filtration with a sieve of 1-mm^2^ mesh size, and the supernatant was designated as the kefir beverage. Recovered grains were used again for the next 24 h fermentation period in fresh cow or soy milk, for a total of 7 fermentations (7 days). A fraction of each of the beverages was used for microbial characterization; the remaining part (designated as the cell-free supernatant (CFS)) was centrifuged, filtered through membranes of 0.22 *μ*m pore size (Sartorius®, Göttingen, Germany), and stored at –80°C until use.

### 2.2. Bacterial Strains

*Escherichia coli* ATCC 11775, *Salmonella enterica* subspecies *enterica* serovar Typhimurium JCM 6977, and *Staphylococcus aureus* ATCC 12600 were used as test microorganisms. They were activated in nutrient broth (Nissui®, Tokyo, Japan) by incubation at 37°C for 24 h. Pathogens were obtained from the JCM collection (Ibaraki, Japan). A 0.5 McFarland suspension of each pathogen was prepared (corresponding to 10^8^ CFU mL^−1^) for antibacterial assays. The strains were kept at −80°C.

### 2.3. Determination of Wet Weight of Kefir Grains and pH and Proximate Analysis of Cow's Milk and Soy Milk Kefir

Kefir grains grown in cow's milk and soy milk were subcultured through seven successive passages in a suitable volume of medium (10% w/v) and incubated at 25°C for 24 h for each fermentation. Kefir grains were washed with sterile water, dried with tissue paper, and weighed on an analytical balance model A200S (Sartorius®). The pH readings were made with a pH metre instrument Docu-pH + metre™ (Sartorius®). Water, fat, and ash content of the kefir samples were measured according to methodology recommended by the Association of Official Analytical Chemists [14]. Total nitrogen was measured by the Kjeldahl method [14] using the ACTAC 1500 Super Kjel digestion and Vapodest distillation system (ACTAC, Tokyo, Japan). Carbohydrates were calculated by subtraction.

### 2.4. Determination of Organic Acid Concentration

Once fermentation was complete, the CFS was evaluated for organic acid content using a Dionex ion chromatography system ICS-1500 equipped with an Ion Pac ICE-AS6 column (9 × 250 mm) (Dionex, Sunnyvale, CA, USA).

### 2.5. Determination of Sugar Concentration

Sugar separation and quantification were carried out through high-performance anion-exchange chromatography (HPAE). Aliquots (20 *μ*L) of CFS were automatically injected into a chromatograph (Thermo Scientific ICS 3000, Dionex Canada Ltd., Oakville, Canada) with a column CarboPac PA1 (250 × 2 mm, 10 mm particle size) preceded by a CarboPac PA1 guard column (50 × 2 mm) with the same packing material. The machinery was equipped with a gradient pump (model SP-5) using pulsed amperometric detection (cell with disposable working gold electrode and pH-Ag/AgCl reference electrode, Dionex/Thermo Scientific). For the elution gradient, ultrapure water (eluent A), 0.25 mol/L NaOH (eluent B), and 1 mol/L acetic acid (eluent C) were used, at a flow rate of 0.25 mL/min at 30°C. The elution gradient started with 40% eluent A and 60% eluent B for 8 min. From 8 min to 30 min, the gradient elution ratio was linearly altered to reach 60% eluent B and 40% eluent C, with the sugars being separated according to their retention times. The transition of final elution conditions to original condition occurred in 1 min. These conditions were maintained for 30 min to remove impurities and to stabilise the base line before applying the next sample. The polypropylene bottles containing the mobile phases were continuously pressurised with nitrogen gas to minimise the development and interaction with carbon dioxide from the air. External calibrations were prepared from standard solutions of glucose, fructose, sucrose, ethanol, lactose, raffinose, and stachyose to identify their respective retention times. Carbohydrate standards (Sigma-Aldrich Co., St. Louis, MO, USA) were also used to identify the different sugars on the basis of their retention times. Chromatogram analysis was carried out using Chromeleon version 5.1 software (Dionex Corporation). The sugar contents were expressed in mg 100 mL^−1^ of sample.

### 2.6. Determination of Concentrations of Amino Acids and Related Compounds

Cow's milk and soy milk kefir CFS were used to determine the composition of amino acids and other related compounds (*γ*-aminobutyric acid (gABA) and taurine (Tau)) by amino acid analyser model L-8000 equipped with an ion exchange #2622 SC column (4.6 × 60 mm) (Hitachi, Tokyo, Japan). The eluted compounds were detected and quantified by a postcolumn ninhydrin labelling method [15].

### 2.7. NAD(P)H Fluorescence-Based GABase Assay

The assay was modified from Passoneau and Lowry [16]. The mastermix consisted of 0.08 U/mL GABase, 5 mM alpha-ketoglutarate, 500 *μ*M NADP, 100 *μ*M DTT, and 4 mM EGTA in 100 mM sodium pyrophosphate, pH 8.6. Aliquots (10 *μ*L) of sample were added to 96-well optical plates (Falcon 353261) followed by 90 *μ*L of mastermix. The reaction was conducted at room temperature for 1 h and measured in a FLUOstar Optima with a 340 nm excitation/450 nm emission filter set (gain, 2103; 10 flashes per well).

### 2.8. Isolation and Purification of Bacteria and Yeasts

One millilitre of each fermented product was diluted in 0.1% (w/v) peptone water. Bacteria and yeasts were enumerated by the surface spread technique [17]. Each diluted sample (100 *μ*L) was spread in four different culture media. Lactic acid bacteria (LAB) were enumerated in MRS (De Man–Rogosa–Sharpe medium) agar (Difco®, Le Pont-de-Claix, France) and GAM (Gifu Anaerobic Medium) agar (Nissui®, Tokyo, Japan). Acetic acid bacteria (AAB) were enumerated in glucose, yeast extract calcium carbonate (GYC) agar (5% glucose (Nacalai Tesque®, Kyoto, Japan); 1% yeast extract (Difco); 0.5% calcium carbonate (Nacalai Tesque); 0.03% bromocresol purple (Wako, Osaka, Japan); 2% agar (Nacalai Tesque)) with 0.01% (w/w) cycloheximide (Nacalai Tesque). Yeasts were enumerated in Potato dextrose agar (PDA) (Eiken Chemical Co., Ltd., Tochigi, Japan) agar with 0.01% (w/w) chloramphenicol (Dr. Ehrenstorfer GmbH®, Augsburg, Germany). After spreading, the GYC plates were incubated at 30°C for 48 h, the MRS and GAM plates were incubated in an anaerobic incubator at 30°C for 72 h, and the PDA plates were incubated at 30°C for 48 h. Then, the best dilution was chosen to obtain mean colony-forming units (on every plate, 30 to 300 CFU was the best dilution), and single colonies for isolation and identification.

### 2.9. Identification of Bacteria and Yeasts by Sequencing the Ribosomal RNA Gene

The bacterial and yeast genomic DNA were extracted from pure cultures. Standard genetic techniques were used, essentially as described by Sambrook and Russell [18]. Hypervariable regions of the 16/28S ribosomal RNA gene (rRNA) were sequenced to identify genotypic characteristics of representative bacteria (V1–V3) and yeasts (D1/D2). The forward primer for bacteria was 7F (5′- AGAGTTTGATYMTGGCTCAG-3′), and the reverse primer was 1510R (5′-ACGGYTACCTTGTTACGACTT-3′). The forward primer for yeasts was LSU-D2f (5′-GTGGTAACTTCCATCTAAAGC-3′), and the reverse primer was LSU-D2R (5′-GGTCCGTGTTTCAAGACGG-3′). Forward and reverse primers were provided by Thermo Fisher Scientific® Inc. (MA, USA). A total of 30 *μ*L polymerase chain reaction (PCR) mixture contained 3 *μ*L buffer solution, 2.4 *μ*L dNTPs, 1.5 *μ*L of each primer, 0.15 *μ*L Ex Taq polymerase (Takara, Shiga, Japan), 20.85 *μ*L purified water, and 0.6 *μ*L of the extracted DNA. The PCR amplification was carried out as follows: for bacteria, template DNA was denatured for 2 min at 96°C, followed by 25 cycles of denaturing at 96°C for 15 s, annealing at 50°C for 15 s, and primer extension at 72°C for 1 min 30 s; for yeasts, template DNA was denatured for 2 min at 96°C, followed by 25 cycles of denaturing at 96°C for 15 s, annealing at 50°C for 15 s, and primer extension at 72°C for 40 s. The amplification products were analysed by electrophoresis on 1.0% agarose gels before being sequenced by Eurofins Genomics Co. Ltd. (Tokyo, Japan). Each bacterial sequence datum was used as a query sequence to search for similar sequences using EzBioCloud database (https://www.ezbiocloud.net/). For yeast identification, NCBI database was used (https://www.ncbi.nlm.nih.gov/blast/). The strains identified here showed similarity higher than 99% with type strains for bacteria and similarity of at least 98% for fungal strains. Our sequences showed high similarity with the following strains: *Acetobacter orientalis* strain 21F-2 (accession number BAMX01000004, 99.78%); *Lactococcus lactis* JCM 5805 (BALX01000047, 100%); *Lactobacillus gallinarum* JCM 2011 (BALB01000057, 99.13%); *Lactobacillus kefiri* LMG 9480 (AJ621553, 99.88%); *Lactobacillus nagelii* DSM 13675 (AZEV01000015, 100%); *Lactobacillus plantarum* JCM 1149 (NR117813.1, 99.88%); *Lactobacillus pentosus* strain 124-2 (NR_029133.1, 99.88%); *Kazachstania unispora* CBS398 (KY103682.1, 100%); *Pichia kudriavzevii* CBS5147 (KY104577.1, 98.64%); *Galactomyces candidum* (MG650611.1, 98.08%); *Geotrichum bryndzae* (KP132252.1, 98%); and *Saccharomyces cerevisiae* ATCC18824 (KC881067.1, 100%). Note that *L. plantarum* was not distinguished from *L. pentosus* by 16S sequencing due to being highly phylogenetically close to each other at this level.

### 2.10. Determination of the ACE Inhibitory Activity

ACE inhibitory activity of kefir samples was measured by ACE Kit-WST® (Dojindo Molecular Technologies, Inc., Kumamoto, Japan), according to the manufacturer's instructions [19]. As mentioned in the instructions, blank 1 (all the reagents without sample) and blank 2 (all the reagents without both sample and enzyme) were prepared and measured together with the samples. Additionally, negative controls of each sample (sample + all the reagents without enzyme) were also prepared and measured. ACE inhibition (%) was calculated as follows:

(1) $$\begin{array}{c} \text{ACE inhibition }\%=\left[\frac{\text{A blank }1-\text{A sample}}{\text{A blank }1-\text{A blank }2}\right]\times 100, \end{array}$$

where A blank 1 is the absorbance of the positive control (without samples); A blank 2 is the absorbance of the reagent blank (without addition of enzyme mixtures); and A sample is the absorbance in the presence of kefir. Samples were tested at five concentrations to construct the standard curve for the determination of the IC_50_ value (concentration of inhibitor required to inhibit 50% of the ACE activity). The magnitude of IC_50_ was expressed as *μ*g/mL. Samples were tested in triplicate.

### 2.11. Measurement of Antioxidant Activity

#### 2.11.1. 2,2′-Azino-bis-3-ethylbenzothiazoline-6-sulfonic Acid

ABTS assay was used to determine antioxidant activity. The activity in cow's milk and soy milk kefir samples was determined spectrophotometrically at 405 nm following the procedure of De Gobba et al. [20]. The addition of antioxidant compounds reduces the ABTS cations, thus causing reagent decolourisation, which is measurable spectrophotometrically, depending on the antioxidant type and concentration as well as the reaction time. The ABTS values were expressed as *μ*mol TE/g. To determine the ABTS radical scavenging activity, a standard curve was constructed with 0–300 *μ*mol of Trolox. Each standard and sample were tested in triplicate.

#### 2.11.2. Oxygen Radical Absorbance Capacity (ORAC) Assay

An ORAC assay was conducted according to the method of Dávalos et al. [21] with slight modification. Briefly, 20 *μ*L of either antioxidant (Trolox) or sample and 120 *μ*L of fluorescein were placed in a well of a black 96-well microplate, and the mixture was preincubated at 37°C for 15 min. Then, 60 *μ*L of AAPH (2,2′-azobis(2-amidino-propane) dihydrochloride) solution was added rapidly, using a multichannel pipet (Thermo Fisher Scientific, Waltham, MA). Finally, the plate was shaken for 30 s, and the fluorescence was recorded using the Infinite 200 Scan Multimode Plate Reader (Tecan®, Austria) every minute for 80 min at 37°C. The excitation and emission wavelengths were 485 and 520 nm, respectively. The final ORAC values were expressed as *μ*mol TE/g sample. To determine the antioxidant activity, a standard curve was constructed with 0–300 *μ*mol of Trolox.

### 2.12. Assay for Ferrous Ion-Chelating Ability

Ferrous ion-chelating ability was determined according to the method of Decker and Welch [22]. Fermented and unfermented samples were separately mixed with 0.1 ml of a 2 mM solution of FeCl2 and 0.2 ml of 5 mM ferrozine. The mixture was then shaken and left to stand at room temperature for 10 min, before the absorbance was measured at 562 nm. To determine the chelating effects, a standard curve was constructed with EDTA at concentrations of 0–0.1 mg mL^−1^.

### 2.13. Measurement of Antibacterial Activity

The inhibitory activity of CFS from fermented and unfermented cow's milk and soy milk against *E. coli* ATCC 11775, *S.* Typhimurium JMC 6977, and *S. aureus* ATCC 12600 was tested. One millilitre of each sample was inoculated with 10 *μ*L of a 0.5 McFarland standard pathogen suspension, uniformly mixed and incubated at 37°C for 48 h. Pathogen growth was detected by assessing turbidity. To determine bacteriostatic/bactericidal activity of the samples, aliquots of 100 *μ*L of each sample were subcultured in nutrient agar (Nissui®) dishes and counted after incubating at 37°C for 24 h. Each test was performed in duplicate.

### 2.14. Statistical Analysis

The results of the independent assays are presented as mean ± standard deviation. Comparisons among replicates in every group were made using the Tukey test in the Statgraphics Plus 5.1® software. All the experiments were performed at least in duplicate.

## 3. Results

### 3.1. Chemical Analysis

Cow's milk and soy milk were fermented by kefir grains. After seven consecutive fermentations, pH decreased to 4.26 ± 0.10 (cow's milk kefir, unfermented pH 6.61) and 4.40 ± 0.05 (soy milk kefir, unfermented pH 6.63) (Table 1). The grains in both the kefirs increased in biomass compared to their initial weight (a 1.58- and 3.15-fold increase for cow's milk and soy milk grains, respectively). Neither ash nor water content was significantly changed by fermentation in either kefir (Table 2). Protein and fat content increased slightly in cow's milk kefir but significantly in soy milk kefir. Carbohydrate concentration decreased in both kefirs due to microbial consumption. The main sugars utilised by microbes were lactose for cow's milk kefir (decrease from 4703 to 3314 mg 100 mL^−1^ during fermentation) and sucrose for soy milk kefir (from 751 to 161 mg 100 mL^−1^) (Table 3). Stachyose and raffinose were maintained at significant concentrations in soy milk kefir, not being assimilated by kefir microorganisms. Sugar content was shown to have decreased more in fermented cow's milk (1.06% and 1.36% of sugar decrease by proximate and HPAE analysis, respectively) than fermented soy milk (0.57% and 0.54% of sugar decrease in proximate and HPAE analysis, respectively) (Tables 2 and 3).

The major organic acid was lactic acid in both fermented kefir beverages (Table 4), with values of 1307 and 698 mg 100 mL^−1^ in fermented cow's milk and soy milk, respectively. The levels of other organic acids varied, with soy milk kefir containing a significantly higher concentration of citric acid (260 mg 100 mL^−1^) than cow's milk kefir (109 mg 100 mL^−1^); in contrast, the acetic acid concentration was higher in cow's milk kefir (135 mg 100 mL^−1^) than soy milk kefir (52 mg 100 mL^−1^).

Analysis for free amino acids showed 9.72 and 33.44 mg 100 mL^−1^ in unfermented cow's milk and soy milk, respectively. These values increased to 20.92 and 36.20 mg 100 mL^−1^ for cow's milk and soy milk kefirs after fermentation, respectively, indicating proteolysis by microorganisms (Table 5). Glutamic acid was the major amino acid in unfermented cow's milk (5.90 mg 100 mL^−1^), and it remained as one of major amino acids after fermentation (6.21 mg 100 mL^−1^). A significant concentration of glutamic acid was also observed for soy milk kefir (16.62 mg 100 mL^−1^), making it the dominant amino acid. Proline was also detected at a high level (5.31 mg 100 mL^−1^) in cow's milk kefir. Interestingly, some amino acid concentrations changed differently between the two types of kefir fermentation. Arginine drastically decreased during soy milk kefir fermentation (from 12.13 mg 100 mL^−1^ to ND), and the concentration of several amino acids (Met, Leu, and Val) decreased significantly in soy milk kefir whereas they increased during the fermentation of cow's milk kefir. Taurine increased after fermentation of both types of milk, and gABA was absent in all samples.

### 3.2. Microbial Analysis

Comparable populations of LAB were detected in both types of kefir (10^8^–10^9^ CFU mL^−1^ and 10^9^ CFU mL^−1^ for cow's milk and soy milk kefirs, respectively) (Table 6). However, yeasts and AAB were found to be present at lower populations in soy milk kefir (10^4^ CFU/mL and ND, respectively) than in cow's milk kefir (10^6^–10^7^ CFU mL^−1^ and 10^6^–10^7^ CFU mL^−1^, respectively). These differences might be due to the lower sugar concentration in soy milk (1.40%) than in cow's milk (4.71%), which in turn suppressed the yeast counts to 2–3 logarithmic orders lower. It is also a severe drawback in soy milk kefir that the second major sugar, stachyose, cannot be metabolised by the kefir microorganisms (Table 3). The lower ethanol concentration detected in soy milk kefir (0.37 versus 1.92 mg 100 mL^−1^ in cow's milk kefir) supports the difference seen in yeast activity, which in turn determines the growth of AAB (Table 6). This not so large difference in ethanol concentration could be explained by higher counts of AAB in cow's milk kefir, which consume a part of ethanol. From the plate counts, LAB, yeasts, and AAB represented 93%–98%, 1%–4%, and 1%–3% of the microorganisms in cow's milk kefir, respectively, while in soy milk kefir almost 100% of microorganisms were LAB.

We determined the microbial species dependent on 16/26S rDNA sequence for representative strains of each colony morphology group (214 isolates were identified in approximately 350 isolates) (Table 6). In both kefir types, *Lactococcus lactis* was identified as the major LAB species (10^7^–10^9^ CFU mL^−1^). As for yeasts, *Kazachstania unispora*, *Pichia kudriavzevii*, and *Galactomyces candidum* were detected on the first day of cow's milk kefir fermentation, but *Saccharomyces cerevisiae* increased to 10^7^ CFU mL^−1^by the 4th day. In contrast, the growth of *S. cerevisiae* in soy milk kefir was weaker (∼10^4^ CFU mL^−1^), indicating that soy milk kefir is not suitable for vigorous growth of this yeast. Similarly, an AAB species, *Acetobacter orientalis*, was only detectable in cow's milk kefir (10^6^–10^7^ CFU mL^−1^), whereas AAB was negligible in soy milk kefir. Some minor species of LAB (*Lactobacillus gallinarum*, *Lactobacillus kefiri*, *Lactobacillus nagelii*, and *Lactobacillus plantarum*/*pentosus*) and yeasts (*Geotrichum bryndzae*) were detected, but their colony counts could not be enumerated due to their scarcity (Table 6).

### 3.3. Functional and Antimicrobial Analysis

ACE inhibitory activity of fermented/unfermented cow's milk and soy milk was measured (Table 7). Unfermented soy milk showed 6.36 times stronger activity than unfermented cow's milk (IC_50_ of 49.50 vs. 7.78 *μ*g/mL for cow's milk and soy milk, respectively). However, both cow's milk and soy milk kefirs showed improved activity, with the cow's milk kefir showing the strongest ACE inhibitory activity (IC_50_ of 0.81 *μ*g/mL).

Soy milk and cow's milk showed inherent antioxidant activity by three methods but at different rates, with the stronger activity overall being in soy milk (Table 8).

Table 9 summarises the antibacterial activity of cow's milk and soy milk kefir. Unfermented cow's milk and soy milk were suitable for growth of pathogens (*E. coli*, *S.* Typhimurium, and *S. aureus*), showing viable counts comparable to nutrient broth (>1.5 × 10^5^ CFU mL^−1^). After fermentation, cow's milk and soy milk kefirs exhibited antibacterial activity against all three pathogens. The bacterium most resistant to kefir was *S. aureus*, which tolerated 25% cow's milk kefir solution and 75% soy milk kefir solution. On the other hand, all tested samples showed bactericidal activity against *E. coli* and *S.* Typhimurium. The lower antibacterial activity of soy milk kefir against *S. aureus* could be due to the low concentration of lactic and acetic acids compared to cow's milk kefir (Table 4).

## 4. Discussion

Milk from mammals (cow, goat, sheep, buffalo, etc.) is the common raw material for kefir grains [3, 23], but the composition of LAB, AAB, and yeasts may change at the species level depending on the grain origin [4, 24]. Growth of these microorganisms makes kefir beverages acidic, as found in this and other studies [6, 25] (Table 1). Significant increases in lactic and acetic acids were observed regardless of the milk type, indicating the vigorous microbial growth supported by consumption of the carbohydrates contained in both cow's milk and soy milk (Table 4). The sugars most consumed were lactose for cow's milk kefir and sucrose for soy milk kefir; thus, the sugar composition could be an important factor selecting the microbes growing in each kefir beverage (Table 3). Notably, a drawback when making kefir from soy milk fermentation could be that the second major sugar, stachyose (44% of total sugars), is not utilised by kefir microorganisms, which might limit the microbial activity in soy milk kefir (Table 3). Our result is not in concordance with that obtained by Baú et al. [6], who reported that soy milk kefir microbes consumed raffinose and stachyose, but this difference might be due to the soy milk origin since they used laboratory-made soy milk in which sugar concentrations were considerably different. The different behaviour of citric acid levels in cow's milk and soy milk kefir (Table 4) indicated that the microbiota can influence the organic acid balance resulting in organoleptic and antimicrobial characteristics of kefirs [2, 13]. Similar results for organic acid content have been reported for cow's milk kefir [13, 25, 26] and soy milk kefir [27, 28].

The total free amino acid concentration for cow's milk kefir differs between the reports. Total free amino acids in this study were 20.92 mg 100 mL^−1^, but three previous reports described variable concentrations from 7 to 76 mg 100 mL^−1^ [29–31]. This difference could arise from a combination of factors such as proteolytic activity, assimilation, and release from the cells [32]. It has also been reported that the nutritional composition of kefir is influenced by milk type, fermentation conditions, kefir grain origin, and storage conditions [4]. The composition of major free amino acids differs between this and other studies [29–31], with the variability likely arising from the aforementioned factors. A significant concentration of Glu was detected in both cow's milk and soy milk kefir in our study, indicating that these beverages could have rich umami-related amino acid properties. Some amino acids, Arg, Met, Leu, and Val, significantly decreased during the soy milk kefir fermentation, and this might have caused the nutritional starvation that limited the microbial growth in soy milk kefir as revealed by the low yeast population and absence of AAB.

There were also variations with other amino acids. Grønnevik et al. [32] detected gABA in cow's milk kefir, levels of which increased during storage; however, we did not detect gABA in either unfermented or fermented cow's milk (measured by both amino acid analyser and enzymatic assay). On the other hand, we detected a comparable concentration of Tau (3.42 mg 100 mL^−1^ in cow's milk kefir) as reported by Irigoyen et al. [30] (0.82–2.66 mg 100 mL^−1^); therefore, some kefir microorganisms were able to synthesise this amino acid (Table 5). Taurine was not detected in unfermented soy milk, but low levels were observed in soy milk kefir (0.19 mg 100 mL^−1^). These results indicate that the pattern of amino acid-related compounds is sometimes robustly and sometimes variably controlled by the presence of kefir microorganisms.

Microbial contents differed greatly between cow's milk and soy milk kefirs (Table 6). Cow's milk kefir showed a typical composition of LAB, AAB, and yeasts (10^8^–10^9^ CFU mL^−1^ LAB and 10^6^–10^7^ CFU mL^−1^ AAB and yeasts) whereas no AAB were counted in soy milk kefir (10^9^ CFU mL^−1^ LAB and 10^4^ CFU mL^−1^ yeasts). *L. lactis* was the major species of LAB in both cow's milk and soy milk kefirs, and a single AAB species, *A. orientalis*, was identified in cow's milk kefir. As mentioned above, the available concentration of sugars in soy milk was low, limiting yeast growth (as seen in limited counts: 10^3^–10^4^ CFU mL^−1^), which in turn meant that conditions were unsuitable for growth of AAB that require the ethanol produced by yeasts [33]. Despite the low yeast counts in soy milk kefir, the levels were within the limits specified for kefir [34]. Notably, the yeast species *P. kudriavzevii, G. candidum,* and *G. bryndzae* were detected only in cow's milk kefir, whereas *S. cerevisiae* and *K. unispora* were commonly found in both cow's milk and soy milk kefirs, possibly due to the different sugar concentrations available. *G. bryndzae* was newly identified in kefir beverage.

The results of some past reports contradict our data, especially regarding yeast populations, and the differences could derive from the setting of fermentation conditions including sugar concentration. Baú et al. [6] used laboratory-made soy milk with a high concentration of sugars, and they found 10^6^ CFU mL^−1^ yeasts, a level 100-fold higher than that in our study. Liu and Chin-Wen [25] investigated kefirs made from reconstituted skimmed milk and laboratory-made soy milk and detected slightly higher viable counts of yeasts (10^5^ CFU mL^−1^) in soy milk kefir than those in our study. Dadkhah et al. [27] and Pourahmad et al. [28] used the same commercial UHT soy milk fortified with 2% sucrose (Maxsoy Company, Karaj, Iran), and both reported considerably higher yeast counts (10^8^–10^9^ CFU mL^−1^). Another report, however, showed extremely low yeast counts (10^2^ CFU mL^−1^) in both cow's milk and soy milk kefirs, indicating that the control of yeast count is sometimes case-dependent and is also influenced by multiple factors other than nutrient availability, e.g., grain origin. As for LAB, it is known that the population in kefir is influenced by the starter grain LAB composition, as reported by Korsak et al. [35] who found that LAB and AAB species are grain-dependent and that the LAB contained in their fermented kefir were almost identical to those in the starter grains. One report using high-throughput 16S rRNA gene pyrosequencing, on the other hand, revealed the predominance of *Lactococcus* in cow's milk kefir, even species initially present at low populations in the grains [24]. Although the detail is still unclear, from this and other studies, the microbiota in the kefir grains partially control those of the fermented kefir beverage, with *L. lactis* being one of the major facilitator LAB in cow's milk and soy milk kefir fermentation. Our analysis clearly showed that it is difficult to maintain the number of AAB and yeasts during the succession in soy milk kefir fermentation; therefore, it is important to always prepare new starter grains to retain the fermentation activity when soy milk kefir is industrially manufactured.

During recent years, a new public consciousness has arisen concerning the need to reduce or replace the use of antibiotics with natural products. There are many reports about the antibacterial activity of cow's milk kefir and the microorganisms contained in it [4, 13, 36]. Cow's milk kefir showed antibacterial activity against different strains of *E. coli* (including EHEC)*, S. aureus, Salmonella* spp.*, Shigella sonnei, Shigella flexneri, Bacillus cereus,* and *Bacillus subtilis* [13]. Carasi et al. [36] found that *Lactobacillus kefiri* isolated from kefir shows antibacterial activity against different enteropathogens. Our results agree with these, showing that fermented cow's milk and soy milk present bactericidal activity against various pathogens (Table 9). This activity could be attributable to the action of lactic and acetic acids [13] and other compounds such as bacteriocins [37].

Probiotics like kefir may change gut microbiota, triggering changes in the profile of short-chain fatty acids, such as acetate [38], that exert anti-inflammatory properties [39]. Luminal lactate promotes enterocyte proliferation and maintains the intestinal barrier [40], and citric acid has been shown to exert antioxidant activity [41]. We, too, detected antioxidant activity in cow's milk and soy milk kefir. Fiorda et al. [42] found that kefir beverages increased their radical scavenging activity from 744.3 to 960.0 *μ*mol Trolox/mL to 807.1 and 1071.0 *μ*mol Trolox/mL in cow's milk-based kefir and soybean-based kefir beverages, respectively. Our results showed that antioxidant activity in cow's milk increased from 667.4 to 1033.5 *μ*mol Trolox/mL to 1403.5–1412.2 *μ*mol Trolox/mL after kefir fermentation. However, soy milk showed stronger inherent antioxidant activity than cow's milk because of the polyphenols and vitamin E it contained; its antioxidant activity was 1469.8–3111.4 *μ*mol Trolox/mL depending on the technique, and levels did not significantly change after kefir fermentation.

It was also found that ACE inhibitory activity was significantly increased in cow's milk (0.81 *μ*g/mL) and soy milk (3.55 *μ*g/mL) kefirs through fermentation (Table 7). According to the World Health Organization, hypertension complications are responsible for millions of deaths every year [7], and the use of ACE inhibitors has marked a great advancement in the treatment of hypertension [43]. Quirós et al. [44] reported that one bioactive peptide obtained from goat's milk fermented with kefir grains showed a very low IC_50_ value of 2 *μ*g/mL. Fermentation by LAB could positively contribute to ACE inhibitory activity, as revealed in this study and as reported by Aihara et al. [45], who described an increase in activity following fermentation of powdered milk with *L. helveticus*. In contrast, Kwon et al. [46] reported a significant decrease in ACE inhibitory activity of soy milk kefir; thus, it would appear that ACE inhibitory activity might be affected either positively or negatively by microbial fermentation.

## 5. Conclusions

We demonstrated that kefir fermentation reduced the content of sugar contained in cow's milk and soy milk, and changed their free amino acid profile. Soy milk kefir showed less microbial diversity than cow's milk kefir, with low yeast counts and an absence of AAB. We report the yeast *G. bryndzae* in cow's milk kefir for the first time. Both cow's milk and soy milk kefir showed significant ACE inhibitory activity, a factor that may be beneficial for people with high blood pressure. Soy milk showed inherent stronger antioxidant activity regardless of the kefir fermentation, although cow's milk kefir improved its antioxidant activity compared to raw cow's milk. Antibacterial activity was proved in both beverages mainly due to high concentrations of organic acids. Soy milk kefir may be a beverage of great interest to vegans or people with disorders like lactose intolerance. Further studies are necessary to elucidate the compounds responsible for ACE inhibitory activity and to corroborate kefir properties in animal models.

## Figures and Tables

**Table 1:** Kefir grain weight and pH during seven consecutive fermentations with cow's milk and soy milk.

Fermentation^*∗*^	pH^*∗∗*^		Grain weight (g)^*∗∗∗*^	
	Cow's milk kefir	Soy milk kefir	Cow's milk kefir	Soy milk kefir
1	4.39	4.47	11.17	12.47
2	4.25	4.35	11.77	15.88
3	4.18	4.39	12.84	18.55
4	4.08	4.36	13.02	20.58
5	4.33	4.48	13.47	23.91
6	4.27	4.40	15.13	27.68
7	4.33	4.37	15.76	31.53
Average +SD	4.26 ± 0.10	4.40 ± 0.05		

^*∗*^Fermentation was carried out at 25°C for 24 h at 10% (w/v) of grain inoculum. ^*∗∗*^Initial pH: 6.61 (cow's milk); 6.63 (soy milk). ^*∗∗∗*^The initial weight was 10 g for both grains.

**Table 2:** Proximate analysis of unfermented and fermented cow's milk and soy milk with kefir grains.

	Unfermented cow's milk (%)	Fermented cow's milk (%)	Unfermented soy milk (%)	Fermented soy milk (%)
Ash	0.75 ± 0.02^a^	0.75 ± 0.03^a^	0.44 ± 0.04^b^	0.40 ± 0.05^b^
Proteins	3.23 ± 0.02^a^	3.25 ± 0.16^a^	4.47 ± 0.06^b^	5.09 ± 0.29^c^
Fat	1.11 ± 0.15 a	1.34 ± 0.34^a^	3.51 ± 0.18^b^	3.87 ± 0.12^c^
Carbohydrates^*∗*^	4.79	3.73	1.21	0.60
Water	90.12 ± 1.26^a^	90.93 ± 1.01^a^	90.37 ± 0.10^a^	90.04 ± 1.05^a^

Values were shown as mean ± standard deviation except carbohydrates (^*∗*^calculated by subtraction). Different lowercase letters in rows indicate that the values are significantly different.

**Table 3:** Sugar and ethanol composition in unfermented and fermented cow's milk and soy milk with kefir grains.

Sugar/ethanol (mg 100 mL^−1^)	Unfermented cow's milk	Fermented cow's milk	Unfermented soy milk	Fermented soy milk
Ethanol	ND	1.92 ± 0.36	ND	0.37 ± 0.02
Glucose	8.78 ± 7.81	34.74 ± 0.69	2.38 ± 0.05	4.01 ± 0.16
Fructose	ND	ND	1.74 ± 1.08	2.60 ± 2.90
Lactose	4703.14 ± 84.86	3314.17 ± 57.81	ND	ND
Sucrose	ND	ND	750.88 ± 12.87	161.44 ± 1.73
Raffinose	ND	ND	38.44 ± 8.14	38.82 ± 5.06
Stachyose	ND	ND	611.08 ± 8.01	658.55 ± 2.85
Total sugar	4711.92 ± 92.67	3349.88 ± 59.85	1404.52 ± 30.15	865.79 ± 12.72

ND: not detected.

**Table 4:** Organic acids in unfermented and fermented cow's milk and soy milk with kefir grains.

Organic acids (mg 100 mL^−1^)	Unfermented cow's milk	Fermented cow's milk (cow's milk kefir)	Unfermented soy milk	Fermented soy milk (soy milk kefir)
Lactic acid	2.0 ± 0.1	1,306.5 ± 111.9	4.1 ± 0.4	697.7 ± 15.6
Acetic acid	20.0 ± 1.4	135.3 ± 15.2	6.2 ± 0.7	51.8 ± 18.0
Pyruvic acid	ND	4.5 ± 7.8	ND	ND
Citric acid	149.0 ± 9.6	109.3 ± 26.6	217.3 ± 14.1	260.2 ± 22.9

ND: not detected. Seven consecutive fermentations were carried out; samples on days 1, 4, and 7 were taken; and organic acids were determined. The values shown are the mean ± standard deviation of these three measurements.

**Table 5:** Amino acids in unfermented and fermented cow's milk and soy milk with kefir grains.

Amino acids (mg 100 mL^−1^)	Unfermented cow's milk	Fermented cow's milk (cow's milk kefir)	Unfermented soy milk	Fermented soy milk (soy milk kefir)
Asp	0.42 ± 0.31^a^	0.03 ± 0.01^a^	1.66 ± 0.01^b^	2.72 ± 0.16^c^
Thr^*∗*^	0.16 ± 0.02^**a**^	1.30 ± 0.33^**b**^	0.20 ± 0.02^**a**^	0.39 ± 0.03^**a**^
Ser	0.14 ± 0.01^**a**^	0.48 ± 0.17^**a**^	0.30 ± 0.02^**a**^	0.54 ± 0.04^**a**^
AspNH_2_	ND	ND	2.16 ± 0.04^**a**^	1.46 ± 0.07^**b**^
Glu	5.90 ± 0.01^**a**^	6.21 ± 0.57^**a**^	8.81 ± 0.03^**a**^	16.62 ± 2.26^**b**^
GluNH_2_	0.25 ± 0.01^**a**^	ND	ND	ND
Gly	0.73 ± 0.04^**a**^	0.63 ± 0.03^**a**^	1.72 ± 0.03^**b**^	2.79 ± 0.10^**c**^
Ala	0.40 ± 0.02^**a**^	1.04 ± 0.13^**a**^	2.10 ± 0.05^**b**^	3.42 ± 0.22^**c**^
Val^*∗*^^∗^	0.22 ± 0.03^**a**^	1.01 ± 0.11^**b**^	0.46 ± 0.01^**a**^	0.09 ± 0.02^**a**^
Cys	Traces	ND	ND	ND
Met^*∗*^	0.02 ± 0. 00^**a**^	0.09 ± 0.01^**a**^	0.30 ± 0.02^**b**^	0.07 ± 0.04^**a**^
Ile^*∗*^	0.08 ± 0.00^**a**^	0.88 ± 0.16^**b**^	0.39 ± 0.02^**a**^	0.10 ± 0.01^**a**^
Leu^*∗*^	0.12 ± 0.01^**a**^	1.04 ± 0.01^**b**^	0.53 ± 0.05^**c**^	0.06 ± 0.02^**a**^
Tyr	0.12 ± 0.01^**a**^	0.53 ± 0.10^**a**^	0.25 ± 0.03^**a**^	1.17 ± 0.11^**b**^
Phe^*∗*^	0.05 ± 0.01^**a**^	0.52 ± 0.01^**b**^	0.57 ± 0.04^**b**^	0.48 ± 0.05^**b**^
Trp^*∗*^	ND	ND	ND	ND
Lys^*∗*^	0.35 ± 0.04^**a**^	0.67 ± 0.01^**a**^	0.77 ± 0.03^**a**^	2.76 ± 0.17^**b**^
His^*∗∗*^	0.07 ± 0.01^**a**^	1.07 ± 0.09^**b**^	0.61 ± 0.01^**c**^	2.08 ± 0.05^**d**^
Arg	0.38 ± 0.02^**a**^	0.10 ± 0.12^**a**^	12.13 ± 0.11^**b**^	ND^**a**^
Pro	0.30 ± 0.02^**a**^	5.31 ± 0.41^**b**^	0.49 ± 0.01^**a**^	1.43 ± 0.07^**c**^
Total	9.72 ± 0.18	20.92 ± 2.43	33.44 ± 0.17	36.20 ± 3.41
Tau	2.59 ± 0.08^**a**^	3.42 ± 0.04^**b**^	ND^**c**^	0.19 ± 0.04^**d**^
gABA	ND	ND	ND	ND

ND: not detected. ^*∗*^Essential amino acids. ^*∗∗*^Essential amino acid for children. Seven consecutive fermentations were carried out; samples on days 1, 4, and 7 were taken; and amino acids were determined. The values shown are the mean ± standard deviation of these three measurements. Different lowercase letters in the rows indicate that the values are significantly different.

**Table 6:** Viable counts for fermented kefir made from cow's milk and soy milk.

Kefir	Fermentation time (d)^1^	Medium	Viable counts of respective colony morphology groups (CFU mL^−1^)	Species of representative strains identified dependent on 16S/26S rDNA^2^
Fermented milk	1	GYC	3.11 ± 0.14 × 10^6^	*Acetobacter orientalis*
		MRS	6.20 ± 0.02 × 10^7^	*Lactococcus lactis*
		MRS	—^3^	*Lactobacillus gallinarum*
		GAM	5.10 ± 0.12 × 10^7^	*Lactococcus lactis*
		GAM	—	*Lactobacillus gallinarum*
		PDA	2.86 ± 0.48 × 10^6^	*Kazachstania unispora*
		PDA	2.04 ± 0.03 × 10^6^	*Pichia kudriavzevii*
		PDA	2.50 ± 0.70 × 10^4^	*Galactomyces candidum*
		PDA	—	*Geotrichum bryndzae*
	4	GYC	6.50 ± 3.53 × 10^7^	*Acetobacter orientalis*
		MRS	9.35 ± 0.21 × 10^8^	*Lactococcus lactis*
		MRS	—	*Lactobacillus kefiri*
		GAM	8.90 ± 0.71 × 10^8^	*Lactococcus lactis*
		PDA	1.90 ± 0.53 × 10^7^	*Saccharomyces cerevisiae*
		PDA	1.91 ± 0.04 × 10^6^	*Kazachstania unispora*
		PDA	4.50 ± 3.53 × 10^4^	*Pichia kudriavzevii*
	7	GYC	4.50 ± 2.12 × 10^7^	*Acetobacter orientalis*
		MRS	1.37 ± 0.07 × 10^9^	*Lactococcus lactis*
		GAM	1.55 ± 0.17 × 10^9^	*Lactococcus lactis*
		PDA	1.90 ± 0.99 × 10^7^	*Saccharomyces cerevisiae*
		PDA	1.89 ± 0.04 × 10^6^	*Kazachstania unispora*
		PDA	2.00 ± 1.41 × 10^4^	*Pichia kudriavzevii*

Fermented soy milk	1	GYC	NC^4^	
		MRS	2.47 ± 0.10 × 10^9^	*Lactococcus lactis*
		GAM	2.42 ± 0.02 × 10^9^	*Lactococcus lactis*
		PDA	1.05 ± 0.32 × 10^4^	*Kazachstania unispora*
	4	GYC	NC	
		MRS	3.15 ± 0.78 × 10^9^	*Lactococcus lactis*
		GAM	4.40 ± 1.27 × 10^9^	*Lactococcus lactis*
		PDA	1.48 ± 0.10 × 10^4^	*Kazachstania unispora*
		PDA	2.60 ± 1.98 × 10^4^	*Saccharomyces cerevisiae*
	7	GYC	NC	
		MRS	3.25 ± 1.34 × 10^9^	*Lactococcus lactis*
		MRS	—	*Lactobacillus nagelii*
		GAM	3.55 ± 0.35 × 10^9^	*Lactococcus lactis*
		GAM	—	*Lactobacillus nagelii*
		GAM	—	*Lactobacillus plantarum*/*pentosus*
		PDA	2.01 ± 0.04 × 10^4^	*Kazachstania unispora*
		PDA	3.35 ± 1.91 × 10^3^	*Saccharomyces cerevisiae*

^1^Seven days of sequential fermentation (each step was conducted at 25°C for 24 h, in total seven times) of cow's milk and soy milk. ^2^ Microorganisms that were isolated and identified but viable counts of them were under the detection limit (<3 × 10^3^ CFU mL^−1^). ^3^NC: not counted nor isolated (˂10^3^ CFU mL^−1^).

**Table 7:** ACE inhibitory activity of unfermented and fermented cow's milk and soy milk with kefir grains.

Samples	IC_50_ (*μ*g/mL)
Unfermented cow's milk	49.50 ± 1.58^a^
Fermented cow's milk (cow's milk kefir)	0.81 ± 0.08^b^
Unfermented soy milk	7.78 ± 0.39^c^
Fermented soy milk (soy milk kefir)	3.55 ± 0.11^d^

Different lowercase letters indicate that the values are significantly different.

**Table 8:** Antioxidant activity of kefir produced from cow's milk and soy milk.

	ABTS assay (*μ*M Eq Trolox mg mL^−1^)	ORAC assay (*μ*M Eq Trolox mg mL^−1^)	Ion-chelating assay (mg EDTA mg mL^−1^)
Unfermented cow's milk	1033.5 ± 8.8^a^	667.4 ± 50.5^a^	0.212 ± 0.002^a^
Fermented cow's milk (cow's milk kefir)	1403.5 ± 18.9^b^	1412.2 ± 31.5^b^	0.060 ± 0.023^b^
Unfermented soy milk	1469.8 ± 33.5^b^	3111.4 ± 607.1	0.185 ± 0.008
Fermented soy milk (soy milk kefir)	1446.8 ± 66.6^b^	3155.7 ± 648.2	0.059 ± 0.014^b^

Different lowercase letters in columns indicate that the values are significantly different.

**Table 9:** Antibacterial activity of kefir against *E. coli*, *S.* Typhimurium, and *S. aureus*.

Type of kefir	CFS conc.	*E. coli* (CFU mL^−1^)^*∗*^	*S.* Typhimurium (CFU mL^−1^)^*∗*^	*S. aureus* (CFU mL^−1^)^*∗*^
Fermented cow's milk (cow's milk kefir)	100% CFS	NG (−)	NG (−)	NG (−)
	75% CFS	NG (−)	NG (−)	NG (−)
	50% CFS	NG (−)	NG (−)	NG (−)
	25% CFS	NG (−)	NG (−)	NG (2.0 × 10^3^)
	Unfermented CFS	G (>1.5 × 10^5^)	G (>1.5 × 10^5^)	G (>1.5 × 10^5^)

Fermented soy milk (soy milk kefir)	100% CFS	NG (−)	NG (−)	NG (−)
	75% CFS	NG (−)	NG (−)	NG (4.0 × 10^2^)
	50% CFS	NG (−)	NG (−)	NG (6.7 × 10^3^)
	25% CFS	NG (−)	NG (−)	G (5.3 × 10^4^)
	Unfermented CFS	G (>1.5 × 10^5^)	G (>1.5 × 10^5^)	G (>1.5 × 10^5^)

Nutrient broth		G (>1.5 × 10^5^)	G (>1.5 × 10^5^)	G (>1.5 × 10^5^)

^*∗*^CFSs were inoculated with 3.5 × 10^5^ CFU of *E. coli*, 3.1 × 10^5^ CFU of *S*. Typhimurium, and 2.0 × 10^5^ CFU of *S. aureus*. G/NG: growth or no growth observed by turbidity. Samples were taken, inoculated, and enumerated in nutrient agar. Viable counts in nutrient agar are shown in parentheses. CFS was considered as bactericidal when no viable counts were observed.

## Data Availability

All data generated or analysed during this study are included in the manuscript.
